# DAILY ASSOCIATIONS BETWEEN STRESSOR CONTROL AND AFFECT VARY AS A FUNCTION OF STRESSOR TYPE

**DOI:** 10.1093/geroni/igac059.502

**Published:** 2022-12-20

**Authors:** Eric Cerino, Susan Charles, Jacqueline Mogle, Laura Klepacz, Jennifer Piazza, Jonathan Rush, David Almeida

**Affiliations:** Northern Arizona University, Flagstaff, Arizona, United States; University of California, Irvine, Irvine, California, United States; The Pennsylvania State University, University Park, Pennsylvania, United States; Department of Psychological Sciences, Northern Arizona University, Flagstaff, Arizona, United States; California State University, Fullerton, Fullerton, California, United States; University of Victoria, Victoria, British Columbia, Canada; The Pennsylvania State University, University Park, Pennsylvania, United States

## Abstract

Perceived control is an important psychosocial correlate of emotional well-being. Using data from the National Study of Daily Experiences (N=1,797, M=55.82 years, SD=10.35, 57.27% Female), we examined how self-reported control over different types of stressors (arguments, avoided arguments, work, home, network) was associated with negative affect (NA) and positive affect (PA). Over 8 consecutive days in waves conducted in ~2008 and ~2017, people reported their daily NA, PA, and control over stressors they had experienced. Within-person associations revealed lower NA on days when stressor control was higher than usual (p<.001), driven by control over arguments, avoided arguments, and work stressors specifically. PA was higher on days when individuals perceived greater control over avoided and actual arguments (ps<.001), but lower on days when individuals perceived greater control over network stressors (p<.01). Results suggest the facilitative role of control over daily stress for emotional well-being depends on the type of stressor experienced.

